# Supplementary material for: Heritability of movie-evoked brain activity and connectivity
Source: eLife. 2026 Aug 4;14:RP106081. doi: 10.7554/eLife.106081 (PMC13436958; doi:10.7554/eLife.106081)
Supplement: Peer review history

# Editor report: eLife Assessment

**DOI:** 10.7554/eLife.106081.3.sa0

**Authors:** Emma Sprooten

**Affiliations:** Donders Institute for Brain, Cognition and Behaviour Netherlands

**Keywords:** Convincing, Valuable

This paper addresses a **valuable** research question on the modest heritability of the brain's response to movie watching, and how heritability varies under different parameters such as regional spatial hyperalignment and BOLD frequency bands. The topic of this paper is of interest to fMRI methodological experts, and potentially to a broader cognitive neuroscience audience, and those with an interest in understanding the heritable sources of individual differences in brain function. Although some of the conclusions could be strengthened by future cross validation studies in independent and larger family-based samples, and through complementary twin/family and SNP-based models, taken altogether, the analyses and results provide **convincing** evidence for the overall conclusions.

# Referee report: Reviewer #1 (Public review)

**DOI:** 10.7554/eLife.106081.3.sa1

Summary:

Gruskin and colleagues use twin data from a movie-watching fMRI paradigm to show how genetic control of cortical function intersects with the processing of naturalistic audiovisual stimuli. They use hyperalignment to dissect heritability into the components that can be explained local differences in cortical-functional topography and those that cannot. They show that heritability is strongest at slower-evolving neural time scales, and more evident in functional connectivity estimates than in response time series.

Strengths:

This is a very thorough paper that tackles this question from several different angles. I very much appreciate the use of hyperalignment to factor our topographic differences and found the relationship between heritability and neural time scales very interesting. The writing is clear and the results are compelling. In general, I don't have many complaints after a couple reads through the manuscript; most of my comments below are relatively minor suggestions and points of clarification.

Weaknesses:

The only "weaknesses" I identified were some points where I think the methods, interpretation, or visualization could be clarified:

On page 16, you compare heritability in functional connectivity (FC) and response time series and find that the heritability effect is larger in FC. In general, I agree with your diagnosis that this is in large part due to the fact that FC captures the covariance structure across parcels, whereas response time series only diverge in terms of univariate time-point-by-time-point differences. Another important factor here is that (within-subject) FC can be driven by intrinsic fluctuations that occur with idiosyncratic timing across subjects and are unrelated to the stimulus (whereas time-locked metrics like ISC and time-series differences cannot, by definition). This makes me wonder how this connectivity result would change if you used intersubject functional connectivity (ISFC) analysis to specifically isolate the stimulus-driven components of functional connectivity (Simony et al., 2016). This, to me, would provide a closer comparison to the ISC and response time series results, and could allow the authors to quantify how much of the heritability in FC is intrinsic versus stimulus-driven. I'm not asking that the authors actually perform this analysis, as I don't think it's critical for the message of the manuscript-but it could be an interesting future direction. As the authors discuss on page 17, I also suspect there's something fundamentally shared between response time series and connectivity as they relate to functional topography (Busch et al., 2021) that drives part of the heritability effect.

The observation that regions with intermediate ISC have the largest differences between MZ, DZ, and UR is very interesting, but it's kind of hard to see in Figure 1B. Is there any other way to plot this that might make the effect more obvious? For example, I could imagine three scatter plots where the x- and y-axes are, e.g., MZ ISC and UR ISC, and each data point is a parcel. In this kind of plot, I would expect to see the middle values lifted visibly off the diagonal/unity line toward MZ. You could even color the data points according to networks like in Figure 3C. (You also might not need to scale the ISC axis all the way to r = 1, which would make the differences more visible.)

On page 9, if I understand correctly, you regress the vector of ISC values across parcels out of the vector of heritability values across parcels and then plot the residual heritability values. Do you center the heritability values (or include some kind of intercept) in the process? I'm trying to understand why the heritability values go from all positive (Figure 2A) to roughly balanced between positive and negative (Figure 2B). Important question for me: How should we interpret negative values in this plot? Can you explain this explicitly in the text? (I also wonder if there's a more intuitive way to control for ISC. For example, instead of regressing out ISC at the parcel/map level, could you go into a single parcel and then regress the subject-level pairwise ISC values out when computing the heritability score?)

On page 4 (line 155), you say "we shuffled dyad labels"-is this equivalent to shuffling rows and columns of the pairwise subject-by-subject matrix combined across groups? I'm trying to make sure your approach here is consistent with recommendations by Chen et al., 2016. Is this the same kind of shuffling used for the kinship matrix mentioned at line 189?

I found panel A in Figure 4 to be a little bit misleading because your parcel-wise approach to hyperalignment won't actually resolve topographic idiosyncrasies across a large cortical distance like what's depicted in the illustration (at the scale of the parcels you're performing hyperalignment within). Maybe just move the green and purple brain areas a bit closer to each other so they could feasibly be "aligned" within a large parcel. Worth keeping in mind when writing that hyperalignment is also not actually going to yield a one-to-one mapping of functionally homologous voxels across individuals: it's effectively going to model any given voxel time series as a linear combination of time series across other voxels in the parcel.

References:

Busch, E. L., Slipski, L., Feilong, M., Guntupalli, J. S., di Oleggio Castello, M. V., Huckins, J. F., Nastase, S. A., Gobbini, M. I., Wager, T. D., & Haxby, J. V. (2021). Hybrid hyperalignment: a single high-dimensional model of shared information embedded in cortical patterns of response and functional connectivity. NeuroImage, 233, 117975. https://doi.org/10.1016/j.neuroimage.2021.117975

Chen, G., Shin, Y. W., Taylor, P. A., Glen, D. R., Reynolds, R. C., Israel, R. B., & Cox, R. W. (2016). Untangling the relatedness among correlations, part I: nonparametric approaches to inter-subject correlation analysis at the group level. NeuroImage, 142, 248-259. https://doi.org/10.1016/j.neuroimage.2016.05.023

Simony, E., Honey, C. J., Chen, J., Lositsky, O., Yeshurun, Y., Wiesel, A., & Hasson, U. (2016). Dynamic reconfiguration of the default mode network during narrative comprehension. Nature Communications, 7, 12141. https://doi.org/10.1038/ncomms12141

Comments on revised version.

The authors have adequately addressed my previous comments. This is a strong contribution: the methods are sophisticated, the statistical treatment is rigorous, and the results are quite interesting/compelling. I'm happy to endorse the revised manuscript as a finalized version.

Just to confirm: The subjects watched all different movies across the two days, right? For a moment I was wondering "are Day 1 and Day 2 repetitions of the same movies?" Given that Day 1 and Day 2 are an organizational feature of several figures, it might be worth making this very explicit in the Methods and reminding the reader in the Results section.

# Referee report: Reviewer #3 (Public review)

**DOI:** 10.7554/eLife.106081.3.sa2

Strengths:

It's sort of novel to study the heritability of movie-watching fMRI data. The methodology the authors used in the paper is also supportive of their findings. Figures are nicely organized and plotted. They finally found that sensory processing in the human brain is under genetic control over stable aspects of brain function (here referring to neural timescale and resting state connectivity).

Weaknesses:

What I am worried about most is the sample size and interpretation of heritability.

(1) Figure 1. I assumed that the authors just calculated the ISC within each group (MZ, DZ, and UR). Of course, you can get different variations between each group. Therefore, there is heritability. Why not calculate ISC across the whole sample, then separate MZ, DZ, and UR?

(2) Heritability scores in the paper are sort of small. If the sample size is small, please consider p-values, which will tell more about the trustworthiness of your heritability.

(3) I don't understand the high-frequency signals in fMRI data. It's always regarded as noise, the band 1 here in particular.

(4) The statement "we show that the heritability of brain activity patterns can be partially explained by the heritability of the neural timescale" should come from Figure 5. However, after controlling for NT, the heritability decreased max. 0.025 in temporal areas. I am not sure this change supports the statement. If the visual cortex is outlined, and combining ISC changes in the visual cortex, I think this would somehow be answered. Instead of delta h2, adding a new model h2 would be obvious to the readers.

(5) Figures 7 and 8, when getting the difference of heritability, please also consider the standard errors of the heritability estimates. Then you can compare across networks/regions.

(6) I think movie VS resting state is a really important result in this paper. However, there is almost no discussion. Discussing this part would be more beneficial for understanding the genetic control over the neuron arousal and excitation circuits.

Comments on revised version.

The whole manuscript has been improved a lot, and the concerns have been clarified.

# Author comment: Author response

**DOI:** 10.7554/eLife.106081.3.sa3

**Authors:** David C Gruskin, Daniel J Vieira, Jessica K Lee, Gaurav Patel

**Affiliations:** Columbia University Irving Medical Center New York United States; New York State Psychiatric Institute New York United States; New York State Psychiatric Institute New York United States; Columbia University Irving Medical Center New York United States

The following is the authors’ response to the original reviews.

**Public Reviews:**

**Reviewer #1 (Public review):**

Summary:

Gruskin and colleagues use twin data from a movie-watching fMRI paradigm to show how genetic control of cortical function intersects with the processing of naturalistic audiovisual stimuli. They use hyperalignment to dissect heritability into the components that can be explained by local differences in cortical-functional topography and those that cannot. They show that heritability is strongest at slower-evolving neural time scales and is more evident in functional connectivity estimates than in response time series.

Strengths:

This is a very thorough paper that tackles this question from several different angles. I very much appreciate the use of hyperalignment to factor out topographic differences, and I found the relationship between heritability and neural time scales very interesting. The writing is clear, and the results are compelling.

We thank Reviewer 1 for their kind words and enthusiastic support of our manuscript.

Weaknesses:

The only "weaknesses" I identified were some points where I think the methods, interpretation, or visualization could be clarified.

(1) On page 16, the authors compare heritability in functional connectivity (FC) and response time series, and find that the heritability effect is larger in FC. In general, I agree with your diagnosis that this is in large part due to the fact that FC captures the covariance structure across parcels, whereas response time series only diverge in terms of univariate time-point-by-time-point differences. Another important factor here is that (within-subject) FC can be driven by intrinsic fluctuations that occur with idiosyncratic timing across subjects and are unrelated to the stimulus (whereas time-locked metrics like ISC and timeseries differences cannot, by definition). This makes me wonder how this connectivity result would change if the authors used inter-subject functional connectivity (ISFC) analysis to specifically isolate the stimulus-driven components of functional connectivity (Simony et al., 2016). This, to me, would provide a closer comparison to the ISC and response time series results, and could allow the authors to quantify how much of the heritability in FC is intrinsic versus stimulus-driven. I'm not asking that the authors actually perform this analysis, as I don't think it's critical for the message of the manuscript, but it could be an interesting future direction. As the authors discuss on page 17, I also suspect there's something fundamentally shared between response time series and connectivity as they relate to functional topography (Busch et al., 2021) that drives part of the heritability effect.

We agree that investigating the heritability of ISFC (or stimulus-driven functional connectivity) would make for a very interesting future direction. Ultimately, we chose to analyze FC (vs. ISFC) profiles to allow for direct comparison with the sizable existing literature on the heritability of FC (such as in our Movie vs. Rest FC analysis) and decided to refrain from analyzing ISFC data in order to keep the present manuscript focused. ISFC analysis of this dataset will be a focus of future work.

(2) The observation that regions with intermediate ISC have the largest differences between MZ, DZ, and UR is very interesting, but it's kind of hard to see in Figure 1B. Is there any other way to plot this that might make the effect more obvious? For example, I could imagine three scatter plots where the x- and y-axes are, e.g., MZ ISC and UR ISC, and each data point is a parcel. In this kind of plot, I would expect to see the middle values lifted visibly off the diagonal/unity line toward MZ. The authors could even color the data points according to networks, like in Figure 3C. (They also might not need to scale the ISC axis all the way to r = 1, which would make the differences more visible.)

We thank R1 for this helpful suggestion- we originally set the y-axis limits to r = 1 in order to facilitate comparison between ISC (Fig. 1B) and FC profile (Fig. 6B) similarity, but we agree that this renders the group differences harder to discern and have updated the plot accordingly (along with thicker lines to enhance readability). We prefer to keep the line plots in the main body as they allow for direct comparison of all three groups on the same plot, but we have included the scatter plot version in Fig. S2 for those who are interested.

(3) On page 9, if I understand correctly, the authors regress the vector of ISC values across parcels out of the vector of heritability values across parcels, and then plot the residual heritability values. Do they center the heritability values (or include some kind of intercept) in the process? I'm trying to understand why the heritability values go from all positive (Figure 2A) to roughly balanced between positive and negative (Figure 2B). Important question for me: How should we interpret negative values in this plot? Can the authors explain this explicitly in the text? (I also wonder if there's a more intuitive way to control for ISC. For example, instead of regressing out ISC at the parcel/map level, could they go into a single parcel and then regress the subject-level pairwise ISC values out when computing the heritability score?).

We indeed included an intercept in this model using MATLAB’s fitlm function. This means that the model estimates the best-fitting line of the following form: heritability_i_=β0+β1ISC_i_ +ε_i_. We agree that the interpretation of these ε_i_ values and alternative approaches to controlling for ISC should be clarified. As such, we have added the following passages to the text:

Methods: “Because the heritability of ISC is constrained by the degree of synchronization in a given area, we also sought to identify areas in which BOLD time courses were more/less heritable than would be expected based on ISC alone by fitting a linear model of the form heritability_i_=β0+β1ISC_i_+ε_i_ and plotting the residuals. Regarding alternative approaches to controlling for ISC, although the heritability model introduced by Ge et al. allows for the inclusion of covariates defined at the subject level (e.g., age), it does not allow for covariates that are defined at the dyad level (e.g., pairwise ISC).”

Results: “Here, negative values in the residual map indicate parcels where heritability is lower than expected based on ISC, while positive values indicate higher-than expected heritability.”

(4) On page 4 (line 155), the authors say "we shuffled dyad labels"- is this equivalent to shuffling rows and columns of the pairwise subject-by-subject matrix combined across groups? I'm trying to make sure their approach here is consistent with recommendations by Chen et al., 2016. Is this the same kind of shuffling used for the kinship matrix mentioned in line 189?

Briefly, shuffling the kinship matrix involved permuting the rows and columns of the matrix in the same manner (also known as the quadratic assignment procedure), whereas shuffling the dyad labels involved random permutations of the three group labels (MZ, DZ, unrelated), which could not be done through matrix operations as the age- and gender matching precluded the use of a complete similarity matrix. However, given concerns raised by Reviewer 2, we have removed our significance claims from this (and similar) sections, which we discuss in more detail in response to Reviewer 2’s weakness A.

(5) I found panel A in Figure 4 to be a little bit misleading because their parcel-wise approach to hyperalignment won't actually resolve topographic idiosyncrasies across a large cortical distance like what's depicted in the illustration (at the scale of the parcels they are performing hyperalignment within). Maybe just move the green and purple brain areas a bit closer to each other so they could feasibly be "aligned" within a large parcel. Worth keeping in mind when writing that hyperalignment is also not actually going to yield a one-to-one mapping of functionally homologous voxels across individuals: it's effectively going to model any given voxel time series as a linear combination of time series across other voxels in the parcel.

We agree that our efforts to present a simplified depiction of hyperalignment may mislead less familiar readers and have amended Fig. 4A according to this suggestion. We have also added text to the methods section (below) to clarify that the outputs of hyperalignment are time series that reflect linear combinations of other voxels’ time series from that parcel.

“This approach independently transforms each subject's data within discrete anatomical parcels into the common space, yielding functionally aligned vertex time series that are calculated as weighted linear combinations of the original time series from all other vertices within that same parcel for that subject.”

(6) I believe the subjects watched all different movies across the two days, however, for a moment I was wondering "are Day 1 and Day 2 repetitions of the same movies?" Given that Day 1 and Day 2 are an organizational feature of several figures, it might be worth making this very explicit in the Methods and reminding the reader in the Results section.

We agree that this would be helpful and have added the following text to the relevant sections:

“All clips were only viewed once by each subject, with the exception of the brief montage which was included at the end of each of the four runs for test-retest purposes.”

“To characterize the heritability of brain responses to complex stimuli, we used 7T fMRI data from 178 HCP Young Adult subjects acquired across two days (using two largely non-overlapping sets of movie stimuli, see Methods)…”

References:

Busch, E. L., Slipski, L., Feilong, M., Guntupalli, J. S., di Oleggio Castello, M. V., Huckins, J. F., Nastase, S. A., Gobbini, M. I., Wager, T. D., & Haxby, J. V. (2021). Hybrid hyperalignment: a single high-dimensional model of shared information embedded in cortical patterns of response and functional connectivity. NeuroImage, 233, 117975. https://doi.org/10.1016/j.neuroimage.2021.117975

Chen, G., Shin, Y. W., Taylor, P. A., Glen, D. R., Reynolds, R. C., Israel, R. B., & Cox, R. W. (2016). Untangling the relatedness among correlations, part I: nonparametric approaches to inter-subject correlation analysis at the group level. NeuroImage, 142, 248259. https://doi.org/10.1016/j.neuroimage.2016.05.023

Simony, E., Honey, C. J., Chen, J., Lositsky, O., Yeshurun, Y., Wiesel, A., & Hasson, U. (2016). Dynamic reconfiguration of the default mode network during narrative comprehension. Nature Communications, 7, 12141. https://doi.org/10.1038/ncomms12141

**Reviewer #2 (Public review):**

Summary:

The authors attempt to estimate the heritability of brain activity evoked from a naturalistic fMRI paradigm. No new data were collected; the authors analyzed the publicly available and well-known data from the Human Connectome Project. The paper has 3 main pieces, as described in the Abstract:

(1) Heritability of movie-evoked brain activity and connectivity patterns across the cortex.

(2) Decomposition of this heritability into genetic similarity in "where" vs. "how" sensory information is processed.

(3) Heritability of brain activity patterns, as partially explained by the heritability of neural timescales.

Strengths:

The authors investigate a very relevant topic that concerns how heritable patterns of brain activity among individuals subjected to the same kind of naturalistic stimulation are. Notably, the authors complement their analysis of movie-watching data with resting-state data.

Weaknesses:

The paper has numerous problems, most of which stem from the statistical analyses. I also note the lack of mapping between the subsections within the Methods section and the subsections within the Results section. We can only assess results after understanding and confirming the methods are valid; here, however, Methods and Results, as written, are not aligned, so we can't always be sure which results are coming from which analysis.

(A) Intersubject correlation (ISC) (section that starts from line 143): "We used nonparametric permutation testing to quantify average differences in ISC for each parcel in the Schaefer 400 atlas for each day of data collection across three groups: MZ dyads, DZ dyads, and unrelated (UR) dyads, where all UR dyads were matched for gender and age in years." ... "some participants contributed to ISC values for multiple dyads (thus violating independence assumptions)"

This is an indirect attempt to demonstrate heritability. And it's also incorrect since, as the authors themselves point out, some subjects contribute to more than one dyad.

Permutation tests don't quantify "average differences", they provide a measure of evidence about whether differences observed are sufficient to reject a hypothesis of no difference.

Matching subjects is also incorrect as it artificially alters the sample; covarying for age and sex, as done in standard analyses of heritability, would have been appropriate.

It isn't clear why the authors went through the trouble of implementing their own nonparametric test if HCP recommends using PALM, which already contains the validated and documented methods for permutation tests developed precisely for HCP data.

The results from this analysis, in their current form, are likely incorrect.

We appreciate that permutation tests do not quantify average differences and intended to write “We used non-parametric permutation testing to quantify [the significance of] average differences…”. Our intention with this analysis was not to demonstrate heritability, but rather to quantify group differences in ISC in a manner that is interpretable for readers who are unfamiliar with *h*^2^ (e.g., “identical twins’ BOLD time courses were 59% more similar than those from pairs of unrelated individuals”) and motivate the formal heritability analysis used later in the paper. Indeed, all of the heritability analyses in this paper leveraged a validated multidimensional heritability method first introduced by Ge et al. (2016) and used by many other investigators since then. Furthermore, we covaried for age and sex at the subject level in all our heritability analyses, and always tested the significance of these heritability values using a validated permutation procedure (the quadratic assignment procedure; Hubert & Schultz, 1976) that respects the non-independence of dyadic data.

Regarding the shuffling procedure used for Figure 1, while PALM is the standard for univariate, subject-level GLMs in the HCP pipeline and can accommodate nested designs (i.e., subjects within families), it is not designed to handle the unique relational dependencies of dyadic ISC analysis (i.e., the same subject contributing to multiple dyads). Although the element-wise resampling approach was the most appropriate approach available, it is known to inflate the false positive rate (Chen et al., 2016; doi:10.1016/j.neuroimage.2016.05.023); given that this analysis was simply meant to motivate our later hypothesis testing heritability analyses, we have removed significance claims from this section of the manuscript. Still, we emphasize that this has no bearing on the validity of our conclusions which were supported by our formal heritability analyses; throughout our paper we have correctly used the appropriate methods to back the stated claims.

(B) Functional connectivity (FC) (section that starts from line 159): Here the authors compute two 400x400 FC matrix for each subject, one for rest, one for movie-watching, then correlate the correlations within each dyad, then compared the average correlation of correlations for MZ, DZ, and UR. In addition to the same problems as the previous analysis, here it is not clear what is meant by "averaging correlations [...] within a network combination". What is a "network combination"? Further, to average correlations, they need to be r-to-z transformed first. As with the above, the results from this analysis in its current form are likely incorrect.

We regret that R2 had difficulty understanding our analysis and have added the following text to the relevant Methods section to clarify our approach:

“For example, there are 16 parcels in the Kong et al. Auditory network and 17 parcels in the Language network, so the FC profile for a given subject’s Auditory-Language network combination consists of the (16 * 17 =) 272 correlation coefficients between all unique pairs of one parcel from each network.”

As we stated in the previous Methods paragraph, “All Pearson *r* values in this and all other analyses were Fisher *z*-transformed before averaging (and converted back to Pearson *r* for visualization)”. Thus, contrary to the reviewer’s assertion, these analyses were performed correctly. Once again, we emphasize that this analysis was not intended to demonstrate heritability, but rather to describe group differences in FC in familiar units.

(C) ISC and FC profile heritability analyses (section that starts from line 175): Here, the authors use first a valid method remarkably similar to the old Haseman-Elston approach to compute heritability, complemented by a permutation test. That is fine. But then they proceed with two novel, ill-described, and likely invalid methods to (1) "compare the heritability of movie and rest FC profiles" and (2) to "determine the sample size necessary for stable multidimensional heritability results". For (1), they permute, seemingly under the alternative, rest and movie-watching timeseries, and (2), by dropping subjects and estimating changes in the distribution.

The (1) might be correct, but there are items that are not clearly described, so the reader cannot be sure of what was done. What are the "153 unique network combinations"? Why do the authors separate by day here, whereas the previous analyses concatenated both days? Were the correlations r-to-z transformed before averaging?

The (2) is also not well described, and in any case, power can be computed analytically; it isn't clear why the authors needed to resort to this ad hoc approach, the validity of which is unknown. If the issue is the possibility that the multidimensional phenotypic correlation matrix is rank-deficient, it suffices that there are more independent measurements per subject than the number of subjects.

Regarding (1), we have clarified in section 2.6 that the 153 unique network combinations reflect each unique pair of 17 Kong networks. All of our analyses, including this one, were performed separately for each day of data collection, as we state throughout the paper and visualize in our figures (although we acknowledge that, on some occasions, we [conservatively] performed FDR-correction on a combined set of p-values, as discussed in our response to K). Given that the null hypothesis for this analysis is that rest FC and movie FC are equally heritable, we are not sure why permuting rest and movie FC matrices would be invalid. All Pearson r values were z-transformed before averaging, as we stated in our paper.

Regarding (2), we included this analysis in response to editorial concerns that our heritability analyses were not sufficiently powered, and we chose this approach because it serves as a simple way to demonstrate the stability of our results at various sample sizes whose validity is self-evident. Furthermore, this sort of subsampling approach has been used many times before in our field (e.g., Marek et al., 2022) and others (e.g., Manyara et al., 2024) to demonstrate the sample-size dependence and stability of statistical effects. We have added text explaining this to the relevant Methods section (2.6).

(D) Frequency-dependent ISC heritability analysis (from line 216): Here, the authors decompose the timeseries into frequency bands, then repeat earlier analyses, thus bringing here the same earlier problems and questions of non-exchangability in the permutations given the dyads pattern, r-z transforms, and sex/age covariates.

We did not use dyadic permutation testing for any of the frequency-dependent ISC analyses; rather, we used the jackknife SEMs to compare heritability across frequency bands and have added an explicit description of this to section 2.7. We have addressed the r-z transform and covariate concerns in previous comments.

(E) FC strength heritability analysis (from line 236): Here, the authors use the univariate FC to compute heritability using valid and well-established methods as implemented in SOLAR. There is no "linkage" being done here (thus, the statement in line 238 is incorrect in this application). SOLAR already produces SEs, so it's unclear why the authors went out of their way to obtain jackknife estimates. If the issue is non-normality, I note that the assumption of normality is present already at the stage in which parameters themselves are estimated, not just the standard errors; for non-normal data, a rank-based inversenormal transformation could have been used. Moreover, typically, r-to-z transformed values tend to be fairly normally distributed. So, while the heritabilities might be correct, the standard errors may not be (the authors don't demonstrate that their jackknife SE estimator is valid). The comparison of h2 between dyads raises the same questions about permutations, age/sex covariates, and r-z transforms as above.

We used jackknife SEs for these analyses to maintain consistency with the multidimensional heritability package used here, which only outputs jackknife SEs. We note that this jackknife approach (and the corresponding multidimensional heritability analysis) was detailed in prior work (Anderson et al., 2021), and that the leave-one-family-out jackknife has a long history of being used to estimate SEs in heritability studies, especially when working with smaller samples (Knapp et al., 1989). We are also not sure what “the comparison of h2 between dyads” means- heritability cannot be compared “between” dyads; rather, it is defined across dyads.

(F) Hyperalignment (from line 245): It isn't clear at this point in the manuscript in what way hyperalignment would help to decompose heritability in "where vs. how" (from the Abstract). That information and references are only described much later, from around line 459. The description itself provides no references, and one cannot even try to reproduce what is described here in the Methods section. Regardless, it isn't entirely clear why this analysis was done: by matching functional areas, all heritabilities are going to be reduced because there will be less variance between subjects. Perhaps studying the parameters that drive the alignment (akin to what is done in tensor-based and deformation-based morphometry) could have been more informative. Plus, the alignment process itself may introduce errors, which could also reduce heritability. This could be an alternative explanation for the reduced heritability after hyperalignment and should be discussed. An investigation of hyperaligment parameters, their heritability, and their co-heritability with the BOLD-phenotypes can inform on this.

To help set up our hyperalignment analyses, we have added text to the introduction explaining how hyperalignment would help to decompose heritability. The description in the Methods section included a reference to Bazeille et al., 2021, in which the hyperalignment method used here is discussed in detail. Still, we have added citations to additional papers (also cited in the Bazeille et al. paper, and elsewhere in our paper) in case that might be helpful. We note that it is not the case that all heritabilities were reduced by hyperalignment- as can be seen in Figs. 4D, 8A, and S15, hyperalignment did increase heritability in some voxels and network combinations. This would be expected under the alternative (albeit unlikely) hypothesis that functional topographies are not heritable, such that topographic variation between related individuals would obscure similarities in their (heritable) topography-independent brain responses. Recognizing that this alternative is unlikely, we believe the main novelty of this analysis comes from the magnitude of the hyperalignment effect (up to 40% of brain-wide heritability) and its spatial pattern (e.g., larger heritability decreases in visual vs. auditory cortex, the opposite of our NT result).

We agree that we would see lower post-hyperalignment heritability if the alignment process itself introduced errors/noise, but this would be deeply surprising as hyperalignment increases ISC by design (and errors/noise could only decrease ISC). To demonstrate this, we have added Figure S7 which shows that (as expected) ISC across all voxels and subject pairs increases after hyperalignment (and that this increase is larger when hyperalignment is performed in larger parcels). Given that hyperalignment increased ISC, and that it is blind to twin status, we are unsure how it could have introduced errors that would have confounded this result.

(G) Relationships between parcel area and heritability (from line 270): As under (F), how much the results are distorted likely depends on the accuracy of the alignment, and the error variance (vs heritable variance) introduced by this.

We agree that alignment accuracy could potentially impact parcel-level differences in how much heritability changes following hyperalignment, and we included the frequency dependent h^2^_residuals_ (controlling for differences in ISC) in Fig. 3 for this reason, as more accurate hyperalignment should result in greater increases in ISC, raising the heritability ceiling. We note that we observe similar relationships between parcel rank and frequency dependent changes in these residualized maps, suggesting that our parcel-level differences are not simply the result of better alignment in more sensory parcels.

(H) Neural timescale analyses (from line 280): Here, a valid phenotype (NT) is assessed with statistical methods with the same limitations as those previously (exchangability of dyads, age/sex covariates, and r-z transforms). NT values are combined across space and used as covariates in "some multivariate analyses". As a reader, I really wanted to see the results related to NT, something as simple as its heritability, but these aren't clearly shown, only differences between types of dyads.

We have addressed the exchangeability, covariates, and r-z transform comments above (in A). As we explained for our FC strength analyses, we are underpowered to evaluate the heritability of unidimensional traits (like the heritability of NT magnitude), and the heritability of a closely-related measure (BOLD turnover magnitude) has already been established in a larger sample of HCP subjects (https://doi.org/10.1152/jn.00402.2022). Still, we agree that more results related to the heritability of NTs would be of interest to our readers. As such, we have added an analysis in section 3.4 quantifying the heritability of multivariate NT topographies and used SOLAR to quantify the heritability of NT magnitudes, with the disclaimer that this and similar analyses are underpowered (hence the large difference in day 1 and day 2 heritability effect sizes). We also removed significance claims for the dyadic NT similarity analysis.

(I) Significance testing for autocorrelated brain maps and FC matrices (from line 310): Here, the authors suddenly bring up something entirely different: reliability of heritability maps, and then never return to the topic of reliability again. As a reader, I find this confusing. In any case, analyses with BrainSMASH with well-behaved, normally distributed data are ok. Whether their data is well behaved or whether they ensured that the data would be well behaved so that BrainSMASH is valid is not described. As to why Spearman correlations are needed here, Mantel tests, or whether the 1000 "surrogate" maps are valid realizations of the data under the null, remains undemonstrated.

We brought up reliability in this section because we show the reliability of our results across the two days of data collection several times in the paper. R2 is correct to point out that BrainSMASH was validated using normally distributed brain maps, and although some of our brain maps contain normally distributed values, others are right skewed (due largely to the fact that many voxels/parcels exhibit low ISC while visual/auditory areas have very high ISC). In preparing our original manuscript, we visualized BrainSMASH’s variogram outputs for one of the most skewed inputs (vertex-wise BOLD time course heritability) and found that the autocorrelation structures of the empirical and null maps were well-matched. We did not include this in the original manuscript as it is not commonplace in the field to report the variograms, see Author response image 1. Furthermore, our use of Spearman (vs. Pearson) correlations renders these distributional differences less relevant, as the Spearman correlation transforms all inputs to a uniform distribution. To empirically check that these distributional differences do not bias our results, we retested the significance of all brain map associations using the spin test (10.1016/j.neuroimage.2018.05.070), an alternative method that does not assume normally distributed inputs, and obtained identical p-values for all analyses (P<.001 in all cases).

**Author response image 1.**

(J) Global signal was removed, and the authors do not acknowledge that this could be a limitation in their analyses, nor offer a side analysis in which the global signal is preserved.

Although we agree that GSR is a contentious preprocessing step for certain analyses, it has explicitly been shown to increase ISC signal-to-noise without compromising FC fingerprints (Graff et al., 10.1016/j.dcn.2022.101087), and it is uncommon to perform ISC analyses with and without GSR. Still, we have added additional text to our Methods section explaining our rationale for using GSR and that this could affect our results. We also re-ran our main analysis (BOLD time course heritability) with and without GSR and found that GSR had little impact on our results; we have included this in our manuscript as Fig. S4.

Specifically, we see that GSR resulted in a slight increase in heritability (average Day 1 h^2^ with/without GSR = .064/.060; Day 2: .068/.061) and almost no effect on the spatial pattern of our results (With GSR/without GSR Spearman ρ = .99, *P*_brainSMASH_ < .001 on both Day 1 and Day 2).

(K) FDR is used to control the error rate, but in many cases, as it's applied to multiple sets of p-values, the amount of false discoveries is only controlled across all tests, but not within each set. The number of errors within any set remains unknown.

We agree that the FDR usage in our original manuscript was inconsistent, in that for two analyses we FDR-corrected p-values from the two days of data collection together (instead of correcting p-values from each day separately and reporting voxels/parcels/etc. that were significant at q<.05 on both days, as in the rest of our analyses). We note that both approaches are more conservative than reporting significant results at q<.05 separately; regardless, to maintain consistency we have updated all analyses such that FDR correction is always performed separately for each day of data collection.

(L) Generally, when studying the heritability of a trait, the trait must be defined first. Here, multiple traits are investigated, but are never rigorously defined. Worse, the trait being analyzed changes at every turn.

Here, we analyze the heritability of movie-evoked BOLD time courses (Figures 1-5) as well as FC profiles (Figures 6-8). We defined FC profiles in our Introduction as an individual’s pattern of pairwise FC strengths (and further detailed how we quantified FC profiles in the relevant Methods section), and believe that “BOLD time course” is a well understood phrase in the field and does not need to be further defined. We also used hyperalignment to decompose the heritability of these traits into topography-dependent and independent portions, and (new to this version) also explicitly quantify the heritability of neural timescales, which we defined as the AUC of the ACF until the first negative ACF value in both the relevant Results and Methods sections.

To make this clearer, we have modified the last paragraph of our Introduction to begin with:

In the present work, we address these questions by analyzing 7T fMRI recordings of a twin sample acquired by the Human Connectome Project (Van Essen et al., 2013) to quantify the heritability of two distinct high-dimensional traits—stimulus-evoked BOLD time courses and functional connectivity profiles—across the cortex.

**Reviewer #3 (Public review):**

Strengths:

It's sort of novel to study the heritability of movie-watching fMRI data. The methodology the authors used in the paper is also supportive of their findings. Figures are nicely organized and plotted. They finally found that sensory processing in the human brain is under genetic control over stable aspects of brain function (here referring to neural timescale and resting state connectivity).

Weaknesses:

What I am worried about most is the sample size and interpretation of heritability.

(1) Figure 1. I assumed that the authors just calculated the ISC within each group (MZ, DZ, and UR). Of course, you can get different variations between each group. Therefore, there is heritability. Why not calculate ISC across the whole sample, then separate MZ, DZ, and UR?

We believe that this question is getting at the difference between pairwise ISC (i.e., correlating one BOLD time course from one subject with that from another subject) and leave-one-subject-out ISC (i.e., correlating one BOLD time course from one subject with the corresponding average time course across all other subjects). We chose to use the pairwise ISC method because it allows us to capitalize on the information contained in the n^2^ pairwise ISC matrix (whereas the other approach averages out meaningful information to yield a n^1^ ISC matrix) and leverage a more sophisticated multidimensional heritability approach. Also, the leave-one-subject-out approach introduces additional issues re: handling family-level data (e.g., should we include a subject’s twin in the leave-one-subject-out average? If so, how should we handle subjects who don’t have a twin in the dataset, as averaging data from different numbers of subjects will lead to different ISC magnitudes? etc.).

(2) Heritability scores in the paper are sort of small. If the sample size is small, please consider p-values, which will tell more about the trustworthiness of your heritability.

We report p-values for heritability throughout our paper (e.g., stating that BOLD time courses are significantly heritable in 99% of parcels in Figure 2), and we believe that the reliability of our spatial maps across days of data collection (also quantified with p-values) further demonstrates the trustworthiness of our results. Finally, as we demonstrate in Figure S5, our sample size is more than sufficient to reliably detect small effects.

(3) I don't understand the high-frequency signals in fMRI data. It's always regarded as noise, the band 1 here in particular.

In addition to driving shared neuronal responses (which are captured in BOLD signal oscillations <.1 Hz or so), movies also elicit shared cardiac, respiratory, and motion responses across participants at higher frequencies. Although we used a relatively conservative denoising approach here, we believe some of these non-neuronal signals are still present in our data; alternatively, it is also possible that these signals reflect “fast” BOLD responses at >.15 Hz (as discussed in 10.1016/j.neuroimage.2021.118658). In any case, the fact that information in this frequency band is considerably less heritable than information in slower frequency bands supports the idea that this band is noisier and suggests that our heritability results are driven by canonical neuronal activity-related BOLD signals.

(4) The statement "we show that the heritability of brain activity patterns can be partially explained by the heritability of the neural timescale" should come from Figure 5. However, after controlling for NT, the heritability decreased max. 0.025 in temporal areas. I am not sure this change supports the statement. If the visual cortex is outlined, and combining ISC changes in the visual cortex, I think this would somehow be answered. Instead of delta h2, adding a new model h2 would be obvious to the readers.

Although the decrease of 0.025 is small, we note that this constitutes around ~50% of BOLD time course heritability in some voxels (seen in comparison to Fig. 4C), and the spatial pattern of this result is quite consistent across days of data collection, indicating its reliability. Furthermore, the whole-brain distributions of results shown in Fig. 5B are clearly skewed towards negative values, indicating that controlling for NT partially reduces (or “explains”) BOLD time course heritability. Still, we agree that showing raw h^2^ values in addition to the difference maps would be helpful for some readers and have added a corresponding supplementary figure (S12) which shows these.

(5) Figures 7 and 8, when getting the difference of heritability, please also consider the standard errors of the heritability estimates. Then you can compare across networks/regions.

We did consider adding standard errors for these heritability estimates, but found that visualizing standard errors for each of the 153 unique network combinations in our heatmaps rendered the visualizations difficult to parse, and given that our hypotheses concerned global (e.g., hyperaligned vs. MSM-aligned) or network-level (e.g., sensory vs. associative) patterns, we focused on calculating standard errors/p-values for these analyses (although we note that dyad-level standard errors can be found in Fig. 6B, where they are clearly marginal compared to the group effects).

(6) I think movie VS resting state is a really important result in this paper. However, there is almost no discussion. Discussing this part would be more beneficial for understanding the genetic control over the neuron arousal and excitation circuits.

We agree that this result was relatively under-explored in our Discussion section and have added additional text (lines 851-855) to connect this result to recent work on arousal-dependent uniqueness of FC.

**Recommendations for the authors:**

**Reviewer #1 (Recommendations for the authors):**

(1) Do the authors have any ideas why we see this hotspot of heritability in pMTG/LOTC? It really jumps out in Figure 1A and Figure 2. The more posterior sensory MT+ area seems to drop when regressing out ISC in Figure 2B, but this pMTG area stays hot. Is there anything special about this kind of multimodal biological motion/action observation / social perception area (Pitcher & Ungerleider, 2021)? I don't think this is necessary to discuss in the manuscript, but I'm curious if the authors have any speculation.

We are not certain as to why BOLD time courses in this parcel are particularly heritable- although this area is associated with biological motion, that particular function tends to be more right lateralized, and here we see nominally higher heritability in the left hemisphere. Per a Neurosynth review (and consistent with the left lateralization), we believe this may have more to do with speech processing, but a more definitive answer will require further investigation.

(2) Page 3, line 127: "More information on these clips"-it might be worth saying a little bit more here just to make sure people understand that these are audiovisual clips, they include language, they're long enough to convey meaningful social and narrative information, etc.

We agree and have added additional details on the clip composition to the relevant methods paragraph.

(3) Figure 1 caption: can you add a sentence reminding readers what's going on with Day 1 and Day 2?

We thank R1 for this suggestion and have added a sentence to this effect at this location.

(4) Page 9, line 379: "although these more associative parcels do not encode a substantial amount of stimulus-specific information"-is this really true? I suspect these association areas still have decent ISCs, even if there are many processing stages downstream of the raw stimulus.

Although these parcels are not the most synchronized by the stimulus, we agree that it is unfair (and vague) to say that they do not encode a substantial amount of stimulus-specific information. We have edited this sentence to make a more specific claim and highlight the relatively lower ISC in these parcels vs. more unimodal sensory areas.

(5) Page 9, line 417: Can you unpack a bit more what you mean by "supra-BOLD frequency band"?

Here, we refer to the fact that BOLD signals resulting from neuronal firing events have frequencies below ~.15 Hz (Josephs and Henson, 1999). We have added additional text and the Josephs and Henson citation to this line to further unpack this point.

(6) Page 18, line 695: This discussion of how attention and gaze might partly shape response time series reminded me of recent work by Borovska & de Haas (2024)-might be worth citing.

We are grateful to R1 for alerting us to this very relevant work and have included a reference to it in our discussion.

(7) Page 19, line 755: I'm not sure I'd describe the hyperalignment results here as a "deleterious effects [on] heritability"-my reading was that hyperalignment allows you to say something more specific about heritability of function by allowing you to effectively factor out heritability effects that reduce to individual differences cortical topography; this seems like a good thing!

We agree that “deleterious” was a poor word choice given its negative connotation, and have edited this sentence to read:

“With this in mind, future studies investigating genetic correlations between brain function and behavioral variables may benefit from hyperalignment, as it can factor out individual-specific cortical topography and thus yield more precise estimates of functional heritability.”

(8) I would love to see a ventral view in some of these plots! Not asking you to recreate the figures, but the ventral temporal cortex is an area of interest for many folks in the movie fMRI space (e.g., Haxby et al., 2011).

We agree that ventral views would be of interest to some readers and have added the corresponding maps for our main results in supplementary figures S3 and S9.

References:

Borovska, P., & de Haas, B. (2024). Individual gaze shapes diverging neural representations. Proceedings of the National Academy of Sciences, 121(36), e2405602121. https://doi.org/10.1073/pnas.2405602121

Haxby, J. V., Guntupalli, J. S., Connolly, A. C., Halchenko, Y. O., Conroy, B. R., Gobbini, M. I., Hanke, M., & Ramadge, P. J. (2011). A common, high-dimensional model of the representational space in human ventral temporal cortex. Neuron, 72(2), 404416. https://doi.org/10.1016/j.neuron.2011.08.026

Pitcher, D., & Ungerleider, L. G. (2021). Evidence for a third visual pathway specialized for social perception. Trends in Cognitive Sciences, 25(2), 100-110. https://doi.org/10.1016/j.tics.2020.11.006

**Reviewer #2 (Recommendations for the authors):**

(1) To address the common core analytical problems listed under (A), (B), (C), (D), (E), and basically throughout the methods:

(a) Conduct permutations with exchangability restrictions to account for the pattern of dyad-relationships as e.g. implemented in PALM.

(b) Control for age and sex covariates as covariates (e.g. as in SOLAR), rather than by matching.

(c) Perform r-to-z transforms when conducting further analyses on correlations that assume normality.

(d) For all analyses that assume normal distributions, e.g. in SOLAR and BrainSMASH, check that this is the case.

We have explained how PALM is not suited for the study of effects that are defined at the dyad level (A), that we controlled for age and sex covariates in all our formal heritability analyses in our original submission (B), that we always performed r-to-z transforms when indicated in our original submission (C), and that our spatial permutation results don’t hinge on distributional differences (D).

(2) Replace SEs derived from kacknife approach with those from SOLAR, or provide a comparison and motivation and/or demonstrate that SEs are correct.

A more thorough explanation of the block jackknife procedure can be found in prior work introducing the multidimensional heritability method used here (Anderson et al., 2021).

(3) Given problem (F & G):

(a) Consider studying the parameters that drive the hyperalignment. They can be included as covariates in heritability analyses, and/or their heritability is of interest to understand the reasons for the heritability reduction post-hyperaligment.

We agree that this would be interesting but the specific parameters that drive hyperalignment are beyond the scope of this study.

(b) Include the alternative explanation of hyperalignment-induced noise in the discussion.

We have added a figure showing that hyperalignment does not increase noise in ISC and explained here why “hyperalignment-induced noise” does not constitute a reasonable alternative explanation for our results.

(4) Add heritability results for NT phenotypes.

We have added heritability analyses for NT topography and (global) NT magnitude, as detailed above.

(5) Motivate global signal removal, and acknowledge this process typically alters results substantially.

We have added an explanation of our rationale for using GSR and shown in this response that it does not in fact substantially alter the results.

(6) Rephrase and/or clarify the following:

(a) "permutations quantify average differences" (under A).

(b) "network combinations" and related analyses (under B & C).

(c) why some analyses are separated per visit/day and others not (C).

(d) methods and reasons for sample size estimation (C).

We have rephrased or clarified all of the above.

**Reviewer #3 (Recommendations for the authors):**

(1) Participants should be recleared. I know HCP 7T data has 184 subjects. How can the authors have 176 twins and 690 unrelated subjects?

As we reported in our Methods section, 178 subjects had complete movie-watching datasets, and 176 subjects had complete movie-watching and resting-state datasets. Of the 178 subjects with complete movie-watching data, we identified 690 age- and sex-matched dyads.

(2) Figure 1. I don't find Figure S1A in Figure S1.

We thank R3 for catching this error- we have amended this reference to read Fig. S1.

(3) I could also suggest putting Figure 1 and Figure 2 together.

We thank R3 for this suggestion- ultimately, we prefer to keep these figures separate to reinforce the difference between our dyadic similarity and formal heritability analyses.
